# Hospital-Level Nurse Communication and 30-Day Readmission in United States Acute Care Hospitals: A Cross-Sectional Centers for Medicare and Medicaid Services Hospital Compare Analysis

**DOI:** 10.3390/nursrep16070222

**Published:** 2026-06-27

**Authors:** Pham Minh Son, Huu Thuan Vo, Vu Thi Xim, Thi Kim Ngan Tran, Thi My Nhung Pham, Thi Anh Nguyen

**Affiliations:** 1 Faculty of Nursing, Nguyen Tat Thanh University, Ho Chi Minh City 70000, Vietnam; sonpm@ntt.edu.vn (P.M.S.); thuanvh@ntt.edu.vn (H.T.V.); vtxim@ntt.edu.vn (V.T.X.); 2 Cho Ray Hospital, Ho Chi Minh City 70000, Vietnam; 3 Faculty of Nursing–Midwifery, Hong Bang International University, Ho Chi Minh City 70000, Vietnam; nganttk2@hiu.vn (T.K.N.T.); nhungptm@hiu.vn (T.M.N.P.)

**Keywords:** communication, patient readmission, quality of healthcare, hospital administration, nursing care, patient discharge, hospital–patient relations, nursing

## Abstract

**Background:** Nurse–patient communication is a nurse-associated, interprofessionally delivered care-process indicator captured by the Hospital Consumer Assessment of Healthcare Providers and Systems (HCAHPS), but its hospital-level association with hospital-wide readmission after structural and case-mix adjustment remains incompletely characterized. **Methods:** We conducted a cross-sectional secondary analysis of publicly available Centers for Medicare and Medicaid Services (CMS) Hospital Compare data. The exposure was the HCAHPS nurse communication composite (April 2024–March 2025), and the outcome was the Hybrid Hospital-Wide All-Cause 30-day Readmission measure (July 2023–June 2024). The primary model adjusted for ownership and US Census region. Robustness was assessed using a six-model hierarchy, including linkage to Provider of Services and HCRIS data to account for teaching intensity, staffing density, and Disproportionate Share Hospital percentage. Additional sensitivity analyses examined survey weighting, survey-volume restriction, lagged HCAHPS scores, HCAHPS-domain specificity, CMS star-rating adjustment, non-linearity, regional interaction, health-system clustering, and alternative functional forms. Findings are interpreted as cross-sectional ecological associations, not causal or predictive effects. **Results:** Among 2844 acute care hospitals, each 10-percentage-point higher patient-perceived nurse communication score was associated with a 0.289 percentage-point lower 30-day readmission rate (95% CI −0.341 to −0.236; *p* < 0.001) in the primary model. The association was consistent across sensitivity analyses, although it was attenuated after additional adjustment for linked structural hospital characteristics. Among HCAHPS domains, discharge information showed the largest association with readmission. These findings indicate a modest but consistent hospital-level association rather than evidence of causality. **Conclusions:** Hospitals with higher patient-perceived nurse communication tended to have lower 30-day readmission rates, although the association was attenuated after adjustment for structural hospital characteristics. Patient-perceived nurse communication may therefore be a useful nurse-associated process indicator for readmission-related benchmarking, although it reflects interprofessional care and residual organizational confounding remains plausible. Longitudinal or interventional studies are needed to determine whether improving nurse communication can reduce readmissions.

## 1. Introduction

Hospital readmissions within 30 days of discharge are widely used as hospital quality and performance indicators and are associated with substantial patient burden and healthcare expenditure [1,2]. Approximately one in five Medicare beneficiaries is readmitted within 30 days of hospitalization in the United States, with the cost of unplanned rehospitalizations estimated at $17.4 billion (Medicare, 2004) [1]. The CMS Hospital Readmissions Reduction Program, established under the Affordable Care Act, penalizes hospitals with higher-than-expected readmission rates, in CMS program framing as a way to incentivize identification of hospital-level factors hypothesized to be amenable to quality improvement [3]; the broader CMS program of public reporting of process measures has been independently linked to outcome improvements at the hospital level [4].

We frame this analysis within Donabedian’s structure–process–outcome model of healthcare quality [5], which provides the guiding rationale for this study: hospital structure (ownership, region, teaching intensity, staffing, and safety-net status) shapes the care processes patients experience, which in turn shape outcomes such as readmission. Within this model, patient-perceived nurse communication is a care-process indicator, but the extent to which such a process reflects nursing care specifically, rather than the broader care team, requires precision. Following Afaneh and colleagues’ concept analysis of surrogate-term proliferation in the literature [6], we distinguish a nurse-associated process, in which nurses participate prominently; a nurse-relevant process, useful for nursing management and benchmarking; and a nursing-sensitive indicator in the strict sense, which is one that demonstrably changes in response to a change in nursing care with other inputs held fixed. Patient-perceived communication, and the discharge teaching it encompasses, is delivered by an interprofessional team (nurses, physicians, pharmacists, case managers, and discharge planners), so we treat it as nurse-associated and, for management purposes, nurse-relevant while reserving nursing-sensitive for a causal property that the present cross-sectional design cannot establish.

Nursing care is a major structural and process contributor to hospital outcomes [5,7]. Nurses provide more direct patient contact than any other healthcare profession, and the nurse–patient communication that arises across admission, treatment, medication counseling, patient education, and discharge planning has been positioned as both a measurable care process and a therapeutic, relational behavior [8,9]. Nurse–patient communication as a nursing care process is conceptually distinct from the broader patient-experience construct measured at the hospital level by HCAHPS, although the latter is the principal instrument available to quantify the former at scale. Suboptimal patient-perceived communication has been associated with preventable readmission in prior individual-level studies; at the patient level, inadequate understanding of medication regimens, follow-up requirements, or warning symptoms has been associated with subsequent emergency-care presentation or rehospitalization [10,11]. The present study uses hospital-level aggregate data and does not test the patient-level mechanism directly.

The HCAHPS survey is a nationally standardized, publicly reported instrument measuring patient-perceived care quality across US hospitals [12,13]. Its nurse communication composite (measure H_COMP_1_A_P) captures the proportion of patients reporting that nurses “always” explained things clearly, listened carefully, and treated patients with courtesy and respect; the composite has reported Cronbach’s α ≥ 0.84 across foundational HCAHPS validation studies [13,14]. Because HCAHPS data are reported at the hospital level alongside clinical outcome metrics, they enable cross-sectional hospital-level (ecological) analyses linking patient-perceived care process to downstream outcomes [15]. Such ecological analyses do not, by themselves, support causal interpretation; they characterize hospital-level associations that may then be tested under longitudinal or interventional designs.

Proximal correlates of the process–outcome association documented in the transitional-care and missed-care literature include discharge instruction quality, medication counseling, and post-discharge self-management adherence [10,16,17,18,19]; the present design does not adjudicate which of these mediate the hospital-level association.

While prior work has examined the association between composite HCAHPS satisfaction and condition-specific readmission rates [20] and between individual HCAHPS items and readmission in patient-level analyses [21], few peer-reviewed studies have specifically isolated the nurse communication composite against the Hybrid Hospital-Wide Readmission measure—the metric introduced by CMS in 2022 to integrate claims with electronic health record data. A further methodological concern is that the CMS Overall Hospital Quality Star Rating, which is frequently used as a covariate in hospital-level studies, itself incorporates readmission performance (approximately 22% of the composite score), creating a part-whole adjustment when used to adjust readmission models. No prior nursing study has explicitly characterized this endogeneity.

This study addresses three linked questions, motivated by a recurring nursing-management need for routinely collected, scalable process indicators amenable to benchmarking [6]. Specifically, we asked: (i) how does patient-perceived nurse communication vary across US acute care hospitals; (ii) is hospital-level nurse communication cross-sectionally associated with 30-day all-cause readmission after adjustment for hospital structural characteristics; and (iii) does inclusion of the CMS Overall Hospital Quality Star Rating produce part-whole adjustment that obscures the substantive nurse-communication estimate?

## 2. Methods

### 2.1. Study Design and Data Source

We conducted a cross-sectional secondary analysis of publicly available datasets from the CMS Hospital Compare program (https://data.cms.gov, accessed on 2 May 2026) CMS Hospital Compare aggregates quality metrics for hospitals participating in Medicare and Medicaid reimbursement at the institutional level. All data are de-identified aggregate institutional-level records; no individual patient data are included. HCAHPS patient-level data are collected by CMS-approved survey vendors using random monthly sampling of recently discharged patients, with standardized mode-of-administration weighting; only hospital-level aggregates are publicly released. Institutional Review Board approval was not required (45 CFR 46.104(d) [4], exempt secondary-use research with de-identified aggregate data).

Data were downloaded from https://data.cms.gov/provider-data (accessed on 2 May 2026): Hospital_General_Information.csv, HCAHPS-Hospital.csv (containing the H_COMP_1_A_P nurse communication composite), and Unplanned_Hospital_Visits-Hospital.csv (containing Hybrid_HWR and component readmission measures). The HCAHPS dataset reflects April 2024 to March 2025; Hybrid_HWR reflects July 2023 to June 2024. These windows partially overlap but are not sequentially ordered: a substantial portion of HCAHPS measurement (October 2024 to March 2025) falls after the close of the Hybrid_HWR window. The two windows result from the CMS public reporting cycle, which releases HCAHPS and Hybrid_HWR on independent schedules; the most recent simultaneous release combines these periods. This design limitation precludes treating nurse communication scores as temporally prior to readmission and precludes causal or predictive interpretation. To address temporal misalignment directly, a lagged sensitivity analysis (planned at the data-inspection stage) used HCAHPS scores from October 2021 to September 2022 (fully preceding Hybrid_HWR by at least nine months); the association was essentially unchanged (β = −0.308; 95% CI −0.361 to −0.255; N = 2357; Supplementary Table S6). For the Model 5 structural-covariate sensitivity (Supplementary Table S7), we additionally linked the CMS Provider of Services Q4 2024 file (NBER mirror; posotherdec2024.csv) by Provider Number to obtain bed count, urban/rural status, and critical-access designation. For the Model 6 structural-confounder sensitivity (Supplementary Table S11), we linked the FY2023 CMS Hospital Provider Cost Report (Healthcare Cost Report Information System, HCRIS, aggregate file CostReport_2023_Final.csv; downloaded from https://data.cms.gov, accessed on 28 May 2026) to obtain teaching intensity (interns/residents full-time equivalent (FTE) per bed), total staffing density (payroll FTE per bed), and the Allowable Disproportionate Share Hospital (DSH) percentage.

### 2.2. Eligibility Criteria and Analytic Sample

**Figure 1:**
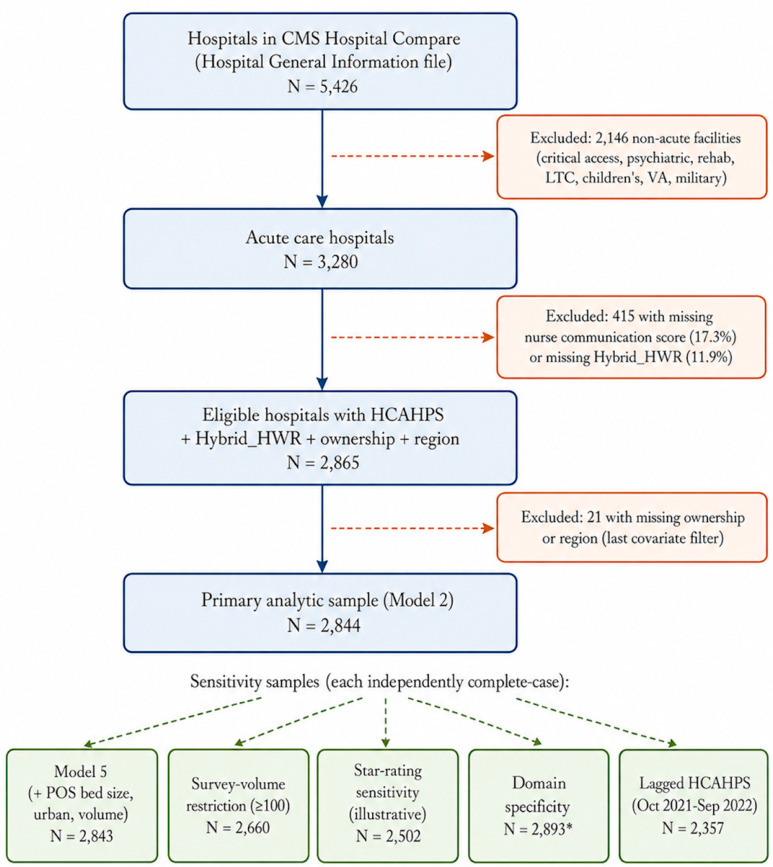
STROBE-style sample flow diagram. Top-down boxes show the primary exclusion pathway from the CMS Hospital Compare general information file (N = 5426) to the primary analytic sample (N = 2844, Model 2). Side boxes show exclusion reasons and counts. Bottom row shows secondary and sensitivity-analytic samples; each is independently complete-case for its specific exposure, reporting window, or linkage requirement, including the new Model 6 HCRIS-linked subsample (N = 2525). * The domain-specificity sample was defined separately for each HCAHPS domain; therefore, hospitals missing the nurse communication score could remain eligible when data for the relevant domain and covariates were complete.

Eligible hospitals were all US acute care hospitals with non-missing values for the primary exposure, primary outcome, and all covariates. Hospitals classified as critical access, children’s, psychiatric, rehabilitation, or long-term acute care were excluded because their patient populations, payment classification, and care processes differ substantially from general adult acute care and because the CMS Hybrid_HWR readmission measure is specified for the general acute care hospital population, while these facility types are reported under separate CMS measures. Figure 1 (STROBE-style sample flow diagram) shows the construction of the primary analytic sample (N = 2844, Model 2) and the sensitivity-analytic subsamples.

### 2.3. Primary Exposure

The primary exposure was the HCAHPS nurse communication composite score (measure H_COMP_1_A_P), defined as the percentage of patients who reported that nurses “always” communicated well. The composite reflects three survey items asking whether nurses explained things in an understandable way, listened carefully to the patient, and treated the patient with courtesy and respect. The score ranges from 0 to 100; higher values indicate a greater proportion of patients reporting consistent high-quality patient-perceived nurse communication. The HCAHPS instrument has been extensively psychometrically validated; the nurse communication composite has reported Cronbach’s α ≥ 0.84 in the foundational CMS/Westat validation studies [13]. In primary analyses, the nurse communication score was treated as a continuous variable and rescaled to 10-percentage-point units. This scale was chosen because a one-point difference is unlikely to represent a meaningful inter-hospital contrast, whereas a 10-percentage-point difference is easier to interpret for nursing-management and quality-improvement audiences. In sensitivity analyses, the score was also categorized into quartiles (Q1–Q4).

### 2.4. Primary Outcome

The primary outcome was the Hybrid Hospital-Wide All-Cause Readmission measure (Hybrid_HWR), reported as a percentage. Hybrid_HWR is a CMS hospital-wide readmission measure adopted for the Hospital Inpatient Quality Reporting Program and publicly released through Hospital Compare. It integrates Medicare claims with electronic health record-derived clinical data elements and replaces the earlier claims-only readmission metric. The measure is risk-adjusted by CMS using patient demographic and clinical characteristics. We used the publicly reported CMS risk-adjusted Hybrid_HWR value as the outcome and did not perform additional patient-level risk adjustment; patient-level demographic, clinical, and socioeconomic data are not contained in the public CMS Hospital Compare files. The CMS Overall Hospital Quality Star Rating was not used as a covariate in the primary or hierarchical models because it incorporates readmission performance directly (~22% of the composite score) and would introduce part-whole adjustment; it was instead included in a separate sensitivity model presented as an endogeneity illustration.

### 2.5. Covariates and Statistical Analysis

The primary model (Model 2) adjusted for two structural hospital characteristics that are present and complete in the primary CMS Hospital Compare files: (i) hospital ownership type (voluntary nonprofit, for-profit, government—reference: nonprofit) because ownership type has been associated with hospital-level patient-experience and clinical outcomes in prior HCAHPS-readmission analyses [20]; and (ii) US Census region (Northeast, Midwest, South, West—reference: South) because substantial unexplained regional variation in 30-day readmission across US Census divisions has been documented [22]. Other structural variables were reserved for sensitivity analyses (Models 3–6) because they required external file linkage.

Six regression specifications were estimated, introduced in numerical order. Model 1 (unadjusted) regressed Hybrid_HWR on the per-10-percentage-point nurse communication score. Model 2 (primary) added hospital ownership and US Census region. Model 3 added log-transformed Hybrid_HWR discharge volume and HCAHPS survey count to address scale-of-operations heterogeneity. Model 4 added US state fixed effects to address geographic clustering at sub-regional scale. Model 5 added log bed count and urban/rural status, linked from the CMS Provider of Services Q4 2024 file by Provider Number (Supplementary Table S7). Model 6 added teaching intensity, staffing density, and DSH percentage, linked from the HCRIS Hospital Provider Cost Report FY2023 (Supplementary Table S11). All six models excluded the CMS Overall Hospital Quality Star Rating; it was tested separately in an endogeneity illustration (Supplementary Table S3). All models used the nurse communication score rescaled to per-10-percentage-point units. Model 2 was prespecified as the primary inferential model because it (i) is estimable in the full analytic sample of N = 2844 hospitals (the inferential population for CMS Hospital Compare cross-sectional analysis), (ii) adjusts only for structural covariates available in the primary CMS Hospital Compare files without external linkage, and (iii) does not condition on staffing density or DSH percentage, which are partly downstream of the nursing care process itself and whose adjustment risks over-control of the very mechanism under study. Model 6 is reported as a structural-covariate sensitivity in the 88.8–linkable subsample (N = 2525); we interpret it as a more conservative bound on the cross-sectional association when measurable structural and safety-net covariates are partialled out, and we report both M2 and M6 estimates throughout (Abstract, Section 3.4, Section 4.3, Section 4.4 and Section 5).

Continuous variables are reported as mean (standard deviation) and median [interquartile range, IQR]; categorical variables are reported as n (%). Pearson correlation quantified the bivariate association. A Breusch–Pagan test assessed heteroskedasticity, with the residuals-versus-fitted diagnostic for the primary model shown in Supplementary Figure S1, and heteroskedasticity-consistent (HC3) robust standard errors were computed alongside ordinary least squares (OLS) estimates. Weighted least squares regression used HCAHPS completed survey count as weights. Influential observations were identified using Cook’s distance (threshold > 4/N), with a sensitivity analysis excluding them. The endogeneity-illustration model added the CMS star rating (complete-case N = 2502; 342 hospitals with missing rating). The survey-volume sensitivity restricted the sample to hospitals with ≥100 completed surveys. The quartile sensitivity replaced the continuous nurse score with quartile categories (Q1–Q4, Model 2 specification). A linearity check added a centered quadratic term in nurse communication to Model 2 (Supplementary Table S8 and Supplementary Figure S2), and a region × nurse communication interaction tested whether the cross-hospital slope varies by US Census region (Supplementary Table S9). Four alternative functional forms—log-outcome OLS, Poisson generalized linear model (GLM) with offset = log (eligible discharges), quasi-binomial GLM with the logit link on the readmission proportion, and beta regression—were estimated under the Model 2 covariate set as a robustness check on the linear-OLS specification (Supplementary Table S10). The hospital is the unit of analysis; CMS aggregates patient responses before public release, so within-hospital patient-level clustering is not observable. Within-system correlation was further addressed by linking the AHRQ Compendium of U.S. Health Systems, 2023 [23], by Medicare CCN and re-estimating the primary model with cluster-robust (CR2) standard errors and a random-intercept multilevel specification (Supplementary Table S12). Model fit is reported as R^2^ and adjusted R^2^. Statistical significance was set at α = 0.05 (two-tailed). Generalized variance inflation factors (VIFs) were computed for multicollinearity. Analyses were performed in R version 4.5.2 (R Foundation for Statistical Computing, Vienna, Austria) [24], using the lme4, sandwich, clubSandwich, lmtest, car, performance, broom, and tidyverse packages. Reporting follows the STROBE checklist for cross-sectional observational studies (Supplementary File S1).

## 3. Results

### 3.1. Sample Characteristics

Of 5426 hospitals in the CMS Hospital Compare general information file, 3280 were classified as acute care. After applying exclusions for missing nurse communication score (17.3% of hospitals with the score reported as “Not Available”), missing Hybrid_HWR (11.9%), and missing ownership or region, the final analytic sample comprised **2844 acute care hospitals** (Figure 1; Table 1).

The mean nurse communication score was 77.9% (SD 5.3; range 53–97%); the mean 30-day all-cause readmission rate was 15.0% (SD 0.77; range 11.7–19.3%). The median number of completed HCAHPS surveys per hospital was 507 (IQR 310–832), and the median eligible discharge volume (Hybrid_HWR denominator) was 1038 (IQR 386–2294). Most hospitals were nonprofit (65.7%), located in the South (41.5%), and had an emergency department (93.8%). The CMS Overall Hospital Quality Star Rating was available for 2502 hospitals (88.0%; mean 3.0, SD 1.1). After the primary HCAHPS/Hybrid_HWR completeness filters, 21 additional hospitals were excluded because ownership or region data were missing; excluded hospitals had a slightly higher mean nurse communication score (82.1% vs. 77.9%) but similar ownership and regional distributions.

### 3.2. Bivariate Association

**Figure 2:**
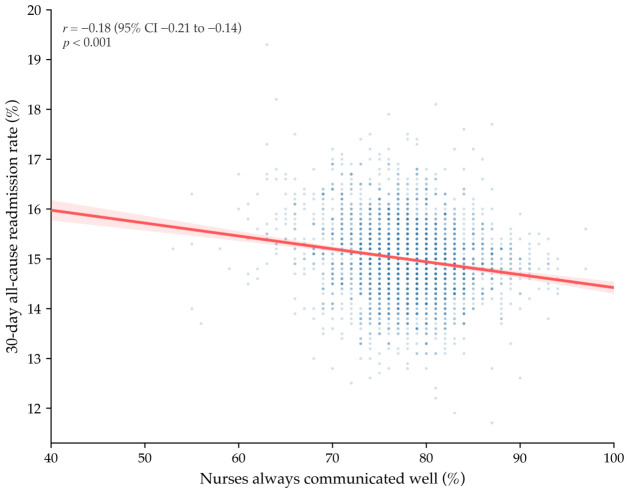
Hospital-level association between patient-perceived nurse communication and 30-day readmission. Scatter plot of HCAHPS nurse communication score versus CMS Hybrid Hospital-Wide 30-day readmission rate across 2844 US acute care hospitals. Each blue point represents one hospital; the red line is the ordinary least-squares fit and the surrounding shaded band its 95% confidence interval. The inset reports the Pearson correlation coefficient (r) with its 95% confidence interval and the associated *p*-value. The association is cross-sectional and hospital-level (ecological) because reporting periods are partially overlapping.

The Pearson correlation between nurse communication score and 30-day readmission rate was r = −0.176 (95% CI −0.212 to −0.140; *p* < 0.001), indicating a statistically significant but modest in magnitude inverse hospital-level (ecological) association—that is, an association observed when the hospital, rather than the individual patient, is the unit of analysis (Figure 2). Given the large sample size, statistical significance alone does not imply a strong association; the correlation should be interpreted as a small effect size at the hospital level. Hospitals in the highest quartile of nurse communication scores (Q4; mean 84.3%) had a mean readmission rate of 14.86% compared with 15.20% in the lowest quartile (Q1; mean 71.3%), an unadjusted mean difference of 0.34 percentage points. This absolute difference is modest at the individual-hospital level; its practical significance depends on hospital scale and on whether the cross-sectional gradient can be translated into a within-hospital intervention effect, which the present design cannot establish.

### 3.3. Multivariable Regression—Primary Model (Model 2)

**Figure 3:**
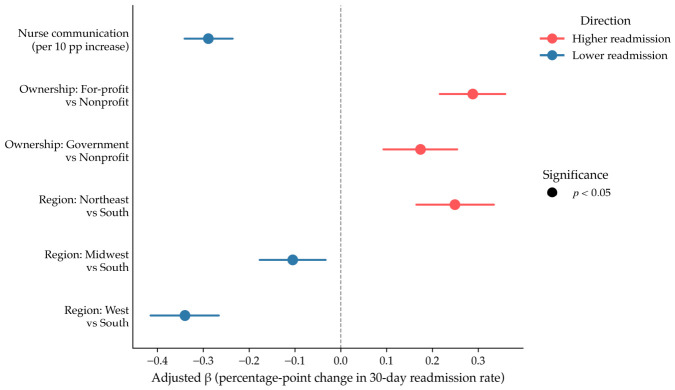
Adjusted hospital-level associations with 30-day readmission. Coefficient plot from the primary Model 2 (adjusted for hospital ownership and US Census region; N = 2844). Each point is the adjusted regression coefficient (β) and the horizontal bar its 95% confidence interval, representing the percentage-point difference in 30-day readmission rate per 10-percentage-point increase in the indicated variable. Point color denotes the direction of the association (blue, lower readmission, β < 0; red, higher readmission, β > 0); filled circles indicate statistical significance (*p* < 0.05); and the dashed vertical line marks the null value of no association. Reference categories: voluntary nonprofit ownership and South region.

In multivariable regression adjusted for hospital ownership type and US Census region, each 10-percentage-point increase in nurse communication score was associated with a 0.289 percentage-point lower 30-day readmission rate (95% CI −0.341 to −0.236; *p* < 0.001) (Table 2; Figure 3). The 95% confidence interval excluded the null. Among covariates, for-profit ownership was associated with higher readmission compared with nonprofit hospitals (β = +0.288; 95% CI 0.216 to 0.359; *p* < 0.001), as was government ownership (β = +0.174; 95% CI 0.093 to 0.254; *p* < 0.001). Compared with Southern hospitals, those in the Northeast had higher readmission rates (β = +0.249; *p* < 0.001), while hospitals in the Midwest (β = −0.105; *p* = 0.004) and West (β = −0.340; *p* < 0.001) had lower rates. The model explained 10.2% of the variance in 30-day readmission rates (R^2^ = 0.102; adjusted R^2^ = 0.100; N = 2844). An R^2^ of approximately 0.10 is within the range typically observed for hospital-level observational analyses of readmission, where a substantial portion of variance is determined by unmeasured patient-level, organizational, and case-mix factors not captured in public hospital quality files; the model is therefore informative about cross-hospital ranking but is not a comprehensive explanatory model of readmission. Generalized VIF values ranged from 1.06 to 1.15, indicating no multicollinearity concern. The Breusch–Pagan test indicated heteroskedasticity (χ^2^ = 37.05, df = 6, *p* < 0.001); HC3 robust standard errors yielded an identical point estimate (β = −0.289; SE = 0.028; *p* < 0.001), confirming that the primary inference is not affected by non-constant variance.

### 3.4. Sensitivity Analyses

**Figure 4:**
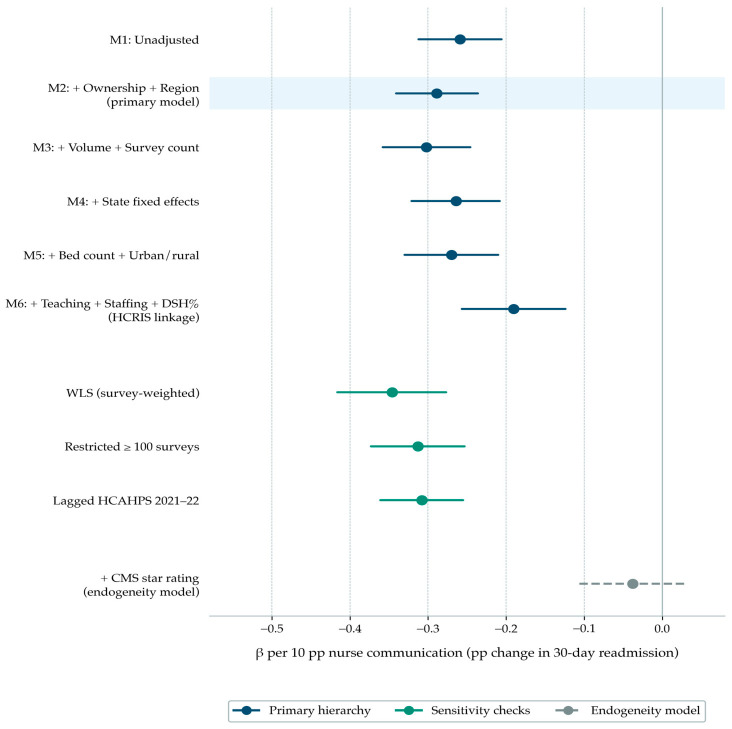
Robustness of the nurse communication–readmission association across model specifications and adjustments. Coefficient estimates represent percentage-point change in 30-day readmission rate per 10-percentage-point increase in nurse communication score. The association remained directionally consistent across the adjustment hierarchy M1–M6; the weighted, restricted, and lagged sensitivity analyses; and the four alternative functional forms. Model 6 (β = −0.190; HCRIS-linked subsample) shows the largest attenuation across the hierarchy; the 26.9% attenuation reported in Section 3.4 is computed relative to the same-subsample Model 5 reference (β = −0.260), which is not plotted. The endogeneity illustration (CMS star rating added) is shown separately as a dashed line to indicate its part-whole adjustment status. Marker and line color denote model class: navy, the primary adjustment hierarchy (Models 1–6); teal, the sensitivity analyses (survey-weighted, restricted to ≥100 surveys, and lagged HCAHPS); and grey, the endogeneity illustration; the light-blue shaded band highlights the primary model (Model 2).

Model adjustment hierarchy. Across the six-model adjustment hierarchy (Supplementary Table S4; Figure 4)—comprising the v1 protocol hierarchy (Models 1–4) and two structural-covariate sensitivity models (Model 5 in the v1 protocol; Model 6 added in response to Reviewer 6.4 during the R1 revision)—the nurse communication association was directionally consistent and statistically significant: M1 unadjusted (β = −0.259; R^2^ = 0.031), M2 primary (β = −0.289; R^2^ = 0.102), M3 adding discharge volume + survey count (β = −0.302; R^2^ = 0.103), M4 adding state fixed effects (β = −0.264; R^2^ = 0.214), M5 adding bed count + urban/rural (linked subsample N = 2843; β = −0.270; Supplementary Table S7), and M6 adding teaching intensity + staffing density + DSH percentage (linked subsample N = 2525; β = −0.190; Supplementary Table S11). The inverse (negative) hospital-level association was not materially attenuated by additional adjustment for hospital volume, survey engagement, or geographic clustering (Models 3–4), although residual confounding cannot be excluded in an observational design; adding the HCRIS structural variables in Model 6, however, produced a 26.9% attenuation relative to the same-subsample Model 5 reference (β = −0.260 → −0.190); within Model 6, total staffing density was protective (β = −0.014 per FTE-per-bed; *p* = 7 × 10^−4^), DSH percentage was positively associated with readmission (β = +0.66 per unit; *p* = 6 × 10^−7^), and teaching intensity was null. The joint contribution of the three HCRIS terms was statistically significant (F(3, 2514) = 11.5; *p* = 1.7 × 10^−7^), indicating that available structural and safety-net covariates account for a substantial confounded component of the cross-hospital association. The 26.9% attenuation is large relative to the kind of “robustness” attenuations typically reported in this literature (single-digit percentage shifts); it is more accurately described as a strong measured-confounding signal than as robustness. The nurse communication coefficient remained statistically significant within Model 6 (β = −0.190; *p* = 2 × 10^−8^), so the residual hospital-level association is not fully reducible to the three HCRIS covariates—but it does not follow that the primary M2 β = −0.289 is the right effect-size summary. Where HCRIS data are available, M6 β = −0.190 is the more defensible point estimate; the primary M2 β = −0.289 should be read as an upper bound that includes ~27% confounding from the measured structural variables and likely further confounding from unmeasured organizational characteristics (Magnet recognition status, registered-nurse-specific staffing skill mix, detailed case-mix complexity).

Quartile graded association. Compared with Q1 (mean nurse score 71.3%), hospitals in Q2, Q3, and Q4 showed progressively lower adjusted readmission rates: Q2 β = −0.193, Q3 β = −0.270, and Q4 β = −0.393 (all 95% CI excluded the null; Supplementary Table S1). The monotonic gradient is consistent with—but does not establish—a graded causal effect; quartile differences could equally reflect uncontrolled structural confounding by hospital characteristics correlated with both HCAHPS performance and readmission.

Weighted regression and influential observations. Weighted least squares (HCAHPS survey count as weights) produced a slightly larger estimate (β = −0.346; 95% CI −0.416 to −0.277), consistent with the primary OLS specification. Excluding 153 influential observations (Cook’s D > 4/N) yielded β = −0.268, confirming the primary finding is not driven by outlier hospitals.

Survey volume restriction. Restricting to 2660 hospitals with ≥100 completed HCAHPS surveys produced a consistent result (β = −0.313; 95% CI −0.373 to −0.253; Supplementary Table S2).

Endogeneity illustration. Adding the CMS Overall Hospital Quality Star Rating to the model attenuated the nurse communication coefficient from −0.289 to −0.038 (*p* = 0.277). The star-rating coefficient was −0.244 (95% CI −0.274 to −0.215; *p* < 0.001), and the Pearson correlation between star rating and readmission rate was −0.380. Because the star rating incorporates readmission performance as approximately 22% of its composite, including it partially regresses the outcome against itself. This specification is presented as a methodological illustration of part-whole adjustment, not as the substantive estimate (Supplementary Table S3).

Linearity and region × communication interaction. A centered quadratic term added to Model 2 (Supplementary Table S8) reached statistical significance (β_2_ = +0.066 per (10 pp)^2^; 95% CI 0.010 to 0.122; *p* = 0.021) but explained ΔR^2^ ≈ 0.002—about 0.2% of additional variance over the linear specification. At N = 2844, this is consistent with random departure from linearity rather than substantive curvature, and the LOWESS smoother (Supplementary Figure S2) tracks the linear OLS fit closely across the empirical range (53–97%), with any deviation concentrated at the extremes where data are sparse. The linear specification is retained as primary for parsimony and interpretability; the quadratic significance should not be over-interpreted given the trivial ΔR^2^. The region × communication interaction was statistically significant (joint F(3, 2834) = 4.6; *p* = 0.003; Supplementary Table S9). The slope was steeper in the West (Δβ vs. South = −0.256; *p* = 0.0003), marginally steeper in the Northeast (Δβ = −0.153; *p* = 0.062), and not different in the Midwest (*p* = 0.21). The marginal Model 2 β = −0.289 is therefore a region-averaged estimate; the underlying gradient is largest in the West.

Functional-form robustness. Four alternative functional forms (log-outcome OLS, Poisson GLM with offset = log (eligible discharges), quasi-binomial GLM with logit link, and beta regression) were all directionally consistent and statistically significant (each *p* < 10^−8^; Supplementary Table S10). On the relative scale, the inverse association corresponds to approximately a 1.7–1.9% reduction in readmission per 10-percentage-point increase in nurse communication score across all four specifications.

### 3.5. Domain Specificity

To assess whether the readmission association is specific to nurse communication or reflects a generic patient-experience signal, we applied the same Model 2 specification to five additional HCAHPS composites: doctor communication (H_COMP_2_A_P), communication about medicines (H_COMP_5_A_P), discharge information (H_COMP_6_Y_P), overall hospital rating (H_HSP_RATING_9_10), and recommend hospital (H_RECMND_DY). The analytic sample for these models was N = 2893 (Supplementary Table S5).

**Figure 5:**
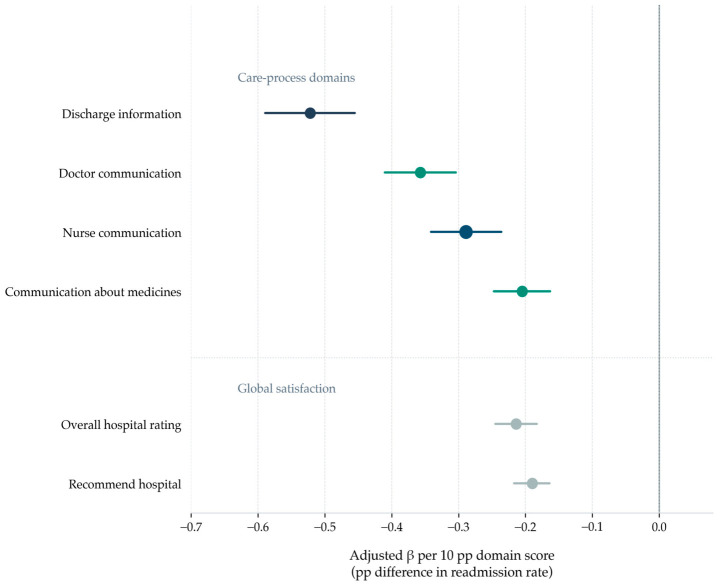
Domain-specific HCAHPS associations with 30-day readmission. Adjusted associations between six HCAHPS domains and 30-day readmission using the same ownership- and region-adjusted Model 2 specification. The care-process panel groups nurse communication, doctor communication, communication about medicines, and discharge information (HCAHPS-canonical care-process composites). The global-satisfaction panel groups overall hospital rating and recommend hospital. Each point is the adjusted regression coefficient (β) per 10-percentage-point increase in the domain score, with the horizontal bar showing its 95% confidence interval and the vertical line marking the null value of no association. Marker color distinguishes the domains: nurse communication—the primary exposure—is highlighted in navy blue with a larger marker; the other care-process domains (discharge information, doctor communication, and communication about medicines) are shown in slate and teal; and the global-satisfaction domains are shown in grey. Discharge information showed the largest cross-sectional association (β = −0.522 per 10 pp); nurse communication ranked third.

Among the six domains, the cross-sectional ranking by absolute β was: discharge information (β = −0.522; 95% CI −0.589 to −0.455; R^2^ = 0.129), doctor communication (β = −0.357), nurse communication (β = −0.289), overall hospital rating (β = −0.214), communication about medicines (β = −0.205), and recommend hospital (β = −0.190) (Figure 5). The two strongest associations belong to care-process domains (discharge information and doctor communication); the two global-satisfaction composites (overall hospital rating and recommend hospital) ranked among the lower three. The pattern is consistent with—but does not demonstrate—care-process specificity over a purely global satisfaction signal; a more definitive test would require a longitudinal design with within-hospital change in the care-process score. Discharge information is a nurse-relevant and frequently nurse-coordinated process but is not exclusively nurse-delivered: physicians, pharmacists, case managers, and discharge planners also contribute. The nurse communication row uses the primary Model 2 complete-case sample (N = 2844) for consistency with Table 2 and the cross-domain ranking; the other five domain rows use the larger N = 2893 (49 additional hospitals with a non-missing non-nurse domain score but a missing nurse communication composite).

## 4. Discussion

### 4.1. Principal Findings

In this cross-sectional ecological analysis of 2844 US acute care hospitals using current CMS Hospital Compare data, higher patient-perceived nurse communication was associated with lower 30-day all-cause readmission. The association was monotonically graded across communication quartiles and was robust across a six-model adjustment hierarchy, weighted regression, survey-volume restriction, structural-covariate sensitivity through Model 6, a lagged HCAHPS reporting window, four alternative functional forms, and a centered-quadratic linearity check. Among six HCAHPS domains tested, the largest cross-sectional association was for discharge information, with nurse communication third by absolute coefficient.

### 4.2. Interpretation and Mechanisms

Clinical magnitude anchor. A 10-percentage-point inter-hospital gradient in nurse communication corresponds, in the primary M2 linear specification, to a 0.289 absolute percentage-point lower readmission rate (95% CI −0.341 to −0.236). On the relative scale (log-outcome OLS sensitivity, Supplementary Table S10), this maps to approximately a 1.9% relative reduction at the mean readmission rate of ~15.0%. For a single hospital with ~1000 eligible Medicare discharges per year, a 10-pp HCAHPS gradient would correspond to ≈3 fewer readmissions per year; the empirical Q1 → Q4 nurse-communication gap of ~13 pp would correspond to ≈4 fewer readmissions per year at the same volume. When HCRIS structural data are available, the equivalent M6 estimate is β = −0.190 (≈2 fewer readmissions per year per 10-pp gradient at a 1000-discharge hospital), which is the more defensible per-hospital expectation. These magnitudes are statistically detectable but small at the individual-hospital level and substantial only when aggregated to the ~32 million annual US hospitalizations base. The cross-sectional gradient cannot be translated into a within-hospital intervention effect: the present design supports neither the claim that improving a hospital’s HCAHPS nurse communication score would lower its readmission rate nor the claim that any specific intervention would do so.

Multiple correlate-level pathways have been proposed in the prior literature that are compatible with the observed cross-sectional pattern. Accurate medication counseling by nurses at discharge has been associated with reduced adverse drug events and subsequent rehospitalization in patient-level studies [7,8]; the corresponding HCAHPS domain at the hospital level is the Discharge information composite, which in our domain-specificity analysis showed the largest cross-sectional association (β = −0.522 per 10 pp). Teach-back communication, in which nurses elicit patient demonstration of discharge understanding, has been associated with improved self-management adherence [17,18] and, in a recent systematic review and meta-analysis of teach-back discharge education in heart-failure patients, with reduced readmission [24,25]; this content area overlaps both the Nurse communication (β = −0.289) and Discharge information items at the hospital aggregate level. Trial-level evidence from randomized studies is consistent with the broader correlate: communication interventions at hospital discharge were associated with lower 30-day readmission in a meta-analysis of randomized trials (relative risk 0.69; 95% CI 0.56–0.84) [25,26]. The patient-level mechanisms inferred from these prior studies are not exclusively nursing-coordinated; physicians, pharmacists, case managers, and unit-level discharge planning systems all contribute to the same patient-perceived domain (see Section 4.3). Whether any of these patient-level mechanisms account for the hospital-level association observed here cannot be determined from the present cross-sectional ecological design.

### 4.3. Comparison with Prior Literature

The directional pattern is consistent with—and extends—the foundational work of Boulding and colleagues [19,20], who reported that overall HCAHPS satisfaction was associated with lower condition-specific readmission at the hospital level using 2005–2008 data. The present analysis advances this literature in three respects: it isolates the nurse communication composite rather than aggregate satisfaction, enabling more targeted policy translation; it uses the Hybrid_HWR (introduced 2022), which integrates claims with electronic health record-derived clinical variables; and it uses data from 2023 to 2025, capturing the post-pandemic period during which US nurse staffing patterns and communication practices were substantially altered [26,27].

Not all prior analyses have reported a significant association between HCAHPS nurse communication and readmission. Yang and colleagues [27,28], in a national sample of 4535 hospitals across six clinical conditions using 2014 data, found that staff responsiveness predicted lower condition-specific readmission rates, but nurse communication was not independently significant. The divergence is consistent with differences in exposure operationalization (item-level vs. composite domain), outcome breadth (condition-specific vs. all-cause all-condition Hybrid_HWR), and data period.

The Model 6 linkage to HCRIS supports a substantive interpretation: the cross-sectional nurse communication association reflects both a nurse-associated component and a hospital structural component. We accordingly describe nurse communication as nurse-associated and, for management benchmarking, nurse-relevant but not nursing-sensitive in the strict sense of responding to a change in nursing care with other inputs held fixed—a causal property that the present cross-sectional design cannot establish. Total staffing density was protective (consistent with the broader nurse-staffing–outcomes literature including Aiken and colleagues’ nine-country European study of 30-day surgical mortality [28,29], Griffiths and colleagues’ analysis of missed care [9,10], and Bridges and colleagues’ work on communication climate [10,11]), and DSH percentage was positively associated with readmission (consistent with the safety-net hospital outcomes literature). The nurse communication coefficient attenuated by 27% within the linked subsample. The more transparent interpretation is that a meaningful fraction of the cross-sectional association is confounded with broader organizational capacity and patient-population disadvantage, while the residual association within Model 6 (β = −0.190) is not fully reducible to these three structural variables but is itself likely subject to further unmeasured confounding from Magnet recognition, RN-specific skill mix, and detailed case mix. The regional heterogeneity revealed by the region × communication interaction (the steepest cross-hospital gradient in the West) is itself consistent with this confounding interpretation: regional variation in Magnet penetration, staffing intensity, and case mix could plausibly co-occur with systematically different cross-hospital slopes across Census divisions.

The attenuation of the nurse communication coefficient to non-significance when the CMS star rating is added (β = −0.038; *p* = 0.28) is best understood as part-whole adjustment /over-adjustment rather than de-confounding: the star rating composite incorporates the readmission outcome domain (~22% weight) and three patient-experience composites that share variance with HCAHPS nurse communication. Adjusting for a covariate that includes the outcome and exposure-related components is statistically equivalent to partialling part of the outcome from itself and yields an attenuated coefficient that does not represent residual confounding by unmeasured hospital quality.

### 4.4. Limitations

Several limitations require acknowledgement. First, and most importantly, the reporting periods are not temporally ordered for causal inference. The HCAHPS nurse communication window (April 2024 to March 2025) extends approximately six months beyond the close of the Hybrid_HWR window (July 2023 to June 2024). This is a feature of the simultaneous CMS Hospital Compare release cycle, not a data collection choice. It precludes interpretation of nurse communication as a “predictor” of readmission and limits all inferences to ecological association. The lagged sensitivity (HCAHPS October 2021 to September 2022, fully preceding Hybrid_HWR by at least nine months) supports temporal robustness but is also cross-sectional in design.

Second, despite a six-model adjustment hierarchy with linkage to the CMS Provider of Services file (Model 5; 99.96% match) and the FY2023 HCRIS Hospital Cost Report (Model 6; 88.8% match), important structural confounders remain absent from the publicly available CMS files. Model 6 demonstrates the practical consequence: adding teaching intensity, staffing density, and DSH percentage attenuated the nurse communication coefficient by 26.9%; staffing density and DSH percentage emerged as significant confounders. The patient-perceived nurse communication score may therefore function in part as a proxy for broader organizational quality, workforce capacity, and safety-net status rather than as an independent measure of nursing communication performance per se. Two specific confounders highlighted by reviewers remain unmeasured in the public CMS aggregate files: Magnet recognition status, which requires linkage to the American Nurses Credentialing Center directory; and registered-nurse-specific staffing skill mix, which requires HCRIS Worksheet S-3 Part I (the underlying hospital-level detailed FTE breakdown not included in the aggregate Hospital Provider Cost Report). Linkage to the American Hospital Association Annual Survey, the National Database of Nursing Quality Indicators, or the AHRQ Healthcare Cost and Utilization Project would also support separation of nursing-specific from broader organizational effects.

Third, the unit of analysis is the hospital, not the patient; patient-level confounders—including socioeconomic position, health literacy, primary language, and clinical severity beyond Hybrid_HWR’s internal CMS risk adjustment—are not available in CMS Hospital Compare data. Ecological fallacy may attenuate or inflate hospital-level associations relative to patient-level effects [30]. Common-method variance is also possible because HCAHPS domains are derived from the same patient-experience survey framework and share a common respondent base. Cross-domain associations may therefore partly reflect respondent-level perception, hospital reputation, or broader satisfaction tendencies rather than independent evaluations of distinct care processes. The domain-specificity analysis showed differential associations across domains, with the largest association for discharge information, which argues against a purely uniform global-satisfaction signal; however, it does not eliminate the possibility that common-method variance contributed to the observed cross-domain pattern.

Fourth, hospitals organized within multi-hospital health systems (for example, HCA Healthcare, Ascension, and Kaiser) may share unmeasured organizational characteristics, including system-wide policies, electronic health record platforms, workforce-management practices, and care-coordination protocols, that induce within-system correlation in HCAHPS and readmission outcomes. Because the primary CMS Hospital Compare files contain no system identifier, we linked the Agency for Healthcare Research and Quality (AHRQ) Compendium of U.S. Health Systems, 2023 [23]. Of the 2844 study hospitals, 2822 (99.2%) were matched, and 2520 (88.6%) belonged to one of 581 health systems, the largest of which (HCA Healthcare) contained 146 study hospitals. The 2520 system-affiliated hospitals formed 581 clusters, and the 324 independent or unmatched hospitals were treated as singleton clusters (905 clusters in total). Between-system clustering was substantial: the intraclass correlation was 0.26 in a null model and 0.21 after covariate adjustment, indicating that roughly a quarter of the variance in 30-day readmission lies between systems and that ordinary standard errors understated uncertainty. The nurse communication association nonetheless persisted. Because most hospitals fall in singleton or small clusters that retain nearly full information, the cluster-robust (CR2) standard error increased only modestly, by a factor of 1.6: the 95% confidence interval widened from the range −0.341 to −0.236 to the range −0.375 to −0.202, but the association remained clearly significant (β = −0.289 percentage points per 10-percentage-point higher score; *p* = 3 × 10^−9^), and a random-intercept specification left the point estimate essentially unchanged (β = −0.263; 95% CI −0.317 to −0.208). Re-clustering by the broader corporate parent gave a near-identical interval (−0.377 to −0.201). Within-system correlation therefore widens the confidence interval but leaves the magnitude of the hospital-level association essentially unchanged (Supplementary Table S12). System membership was taken from the 2023 Compendium, the most recent available; affiliations are stable year to year, but a small number of hospitals may have changed system membership before the 2024–2025 outcome window.

Fifth, the linearity check returned a small statistically significant quadratic term but with trivial added variance explained (ΔR^2^ ≈ 0.002), consistent with the LOWESS smoother (Supplementary Figure S2) tracking the linear fit across the empirical range. The linear specification is therefore appropriate for inference; the quadratic-term significance reflects detection power at N = 2844 rather than substantive curvature, and we do not interpret it as evidence of differential responsiveness at high or low ends of the score distribution. Extrapolation beyond the observed range (53–97%) remains unsupported on general grounds.

Sixth, hospitals with incomplete data were excluded at different stages of sample construction. After the primary HCAHPS/Hybrid_HWR completeness filters, 21 additional hospitals were excluded because ownership or region data were missing. Excluded hospitals with available nurse communication scores had a slightly higher mean nurse communication score (82.1% vs. 77.9%) than included hospitals, suggesting limited selection differences. The missingness fraction is low (<1% of acute care hospitals with nurse scores). HCAHPS response rates vary by hospital and by patient subgroup: response patterns vary systematically across patient age, primary language, socioeconomic position, and clinical severity [31], and hospitals with sicker, more socially disadvantaged, or non-English-preferring patient mixes may show systematically different HCAHPS scores for reasons unrelated to actual nursing communication quality.

## 5. Conclusions

Across 2844 US acute care hospitals, higher patient-perceived nurse communication was consistently associated with lower 30-day readmission; the association attenuated after adjustment for structural and safety-net characteristics and is not, by itself, evidence of a causal effect. Because patient-perceived communication is delivered by the broader care team, it is best regarded as a nurse-associated process indicator rather than a nursing-sensitive outcome; it may nonetheless be a useful, routinely reported signal for readmission-related quality improvement, with residual organizational confounding remaining plausible. Establishing whether deliberately improving communication lowers readmission will require within-hospital longitudinal or interventional designs rather than the cross-sectional comparison reported here.

There are implications for nurse managers and unit-based nurse leaders. Patient-perceived nurse communication scores reported in HCAHPS are a routinely collected, scalable hospital-level signal that nurse managers may consider alongside other nursing-relevant indicators when reviewing unit-level performance [6]. The strongest single domain–readmission gradient in the present analysis was for discharge information, which is multidisciplinary in delivery (involving physicians, pharmacists, case managers, and discharge planners) but is frequently nurse-coordinated. Structured teach-back protocols, bedside handover models, and discharge communication curricula are candidate process improvements with prior randomized-trial support for reducing readmission [25,26] and could be evaluated locally; the present cross-sectional evidence does not, however, support an estimate of expected within-hospital effect size from any specific intervention.

There are implications for hospital quality directors. The endogeneity illustration is a practical caution: studies using CMS Hospital Compare data should not include the CMS Overall Hospital Quality Star Rating as a covariate when modeling any outcome domain (readmission, mortality, safety events) that contributes to the star rating composite. Doing so produces part-whole adjustment that obscures rather than de-confounds the substantive estimate. Hospital quality teams using HCAHPS for internal benchmarking can use the nurse communication composite as a routinely available signal, with the caveat that part of the cross-hospital gradient reflects broader organizational quality and safety-net status rather than independent nursing communication performance per se.

There are implications for researchers. The cross-sectional gradient identified here is a candidate hypothesis for prospective testing. Longitudinal evaluation, either within-hospital before–after designs at sufficient temporal separation or cluster-randomized evaluation of specific communication interventions, would be required to convert the present ecological signal into causal evidence. Future linkage to the American Nurses Credentialing Center Magnet directory, to HCRIS Worksheet S-3 Part I for registered-nurse-specific staffing skill mix, to the American Hospital Association Annual Survey, or to the National Database of Nursing Quality Indicators would support a more complete decomposition of the components proposed here (nurse-associated, nurse-relevant, and nursing-sensitive), building on Afaneh and colleagues’ analysis of surrogate-term proliferation in the nursing-sensitive-indicator literature [6].

## Figures and Tables

**Table 1:** Characteristics of the analytic sample (N = 2844 acute care hospitals).

Characteristic	Value
**N hospitals**	**2844**
Nurse communication score, mean (SD), %	**77.9 (5.3)**
—Range, %	53–97
30-day readmission rate (Hybrid_HWR), mean (SD), %	**15.0 (0.77)**
—Range, %	11.7–19.3
HCAHPS completed surveys, median [IQR]	**507 [310–832]**
Eligible discharges (Hybrid_HWR denominator), median [IQR]	**1038 [386–2294]**
**Geographic region, n (%)**	
—Northeast	423 (14.9)
—Midwest	661 (23.2)
—South	1179 (41.5)
—West	581 (20.4)
**Hospital ownership, n (%)**	
—Voluntary nonprofit	1869 (65.7)
—For-profit	571 (20.1)
—Government	404 (14.2)
Hospital overall star rating, mean (SD) *	3.0 (1.1)
Emergency services, n (%)	2667 (93.8)

* Star rating available for 2502 hospitals (88.0%); excluded from the primary and hierarchical models due to part-whole adjustment (see Section 2.4 and Section 3.4). HCAHPS = Hospital Consumer Assessment of Healthcare Providers and Systems; IQR = interquartile range; SD = standard deviation.

**Table 2:** Multivariable linear regression—adjusted associations with 30-day hospital readmission rate (primary Model 2; N = 2844).

Variable	β	95% CI	*p*-Value
Nurse communication score (per 10-percentage-point increase)	−0.289	−0.341 to −0.236	<0.001
Ownership: For-profit (reference: Voluntary nonprofit)	+0.288	+0.216 to +0.359	<0.001
Ownership: Government (reference: Voluntary nonprofit)	+0.174	+0.093 to +0.254	<0.001
Region: Northeast (reference: South)	+0.249	+0.165 to +0.334	<0.001
Region: Midwest (reference: South)	−0.105	−0.177 to −0.033	0.004
Region: West (reference: South)	−0.340	−0.415 to −0.266	<0.001
Model fit	R^2^ = 0.102	Adj. R^2^ = 0.100	N = 2844

β = unstandardized regression coefficient, interpreted as the percentage-point change in 30-day readmission rate for the indicated comparison. All associations are ecological, hospital-level correlations; temporal ordering between exposure and outcome is not established (see Section 2.1). The CMS Overall Hospital Quality Star Rating is excluded from this model because it incorporates readmission performance directly (see Section 2.4 and Supplementary Table S3). Breusch–Pagan *p* < 0.001; HC3 robust SE for the nurse score coefficient yields an identical point estimate of −0.289 (SE = 0.028).

## Data Availability

All primary data analyzed in this study are freely and publicly available at https://data.cms.gov/provider-data/topics/hospitals (CMS Hospital Compare; accessed on 2 May 2026), https://data.cms.gov/provider-summary-by-type-of-service (CMS HCRIS Hospital Provider Cost Report; accessed on 28 May 2026), and https://data.nber.org/cms/pos/csv/2024/posotherdec2024.csv (CMS Provider of Services file, Q4 2024, via the NBER mirror; accessed on 6 May 2026). The R analysis scripts, sibling provenance YAML files, and derived datasets are available from the corresponding author on reasonable request and are deposited in a project repository.

## Supplementary Materials

The following supporting information can be downloaded at: https://www.mdpi.com/article/10.3390/nursrep16070222/s1, Table S1: Quartile Analysis—Nurse Communication Quartile and 30-Day Readmission Rate; Table S2: Survey Volume Restriction—Hospitals with ≥100 Completed HCAHPS Surveys; Table S3: Endogeneity Sensitivity—CMS Star Rating Sensitivity Model (N = 2502); Table S4: Model Adjustment Hierarchy—Nurse Communication Coefficient Across Six Models; Table S5: HCAHPS Domain Specificity—Six Patient-Experience Composites and 30-Day Readmission; Table S6: Lagged HCAHPS Sensitivity—Nurse Communication (October 2021–September 2022) versus Current 30-Day Readmission (Hybrid_HWR July 2023–June 2024); Table S7: Structural Covariate Sensitivity—CMS Provider of Services Linkage (Model 5; N = 2843); Table S8: Linearity Check—Centered Quadratic Term in Model 2 (N = 2844); Table S9: Region × Nurse Communication Interaction (N = 2844); Table S10: Functional-Form Robustness—Five Specifications of the Model 2 Covariate Set (N = 2844); Table S11: Model 6 Structural-Confounder Sensitivity—HCRIS Linkage (N = 2525); Table S12: Sensitivity of the Nurse Communication–Readmission Association to Health-System Clustering (AHRQ Compendium of U.S. Health Systems, 2023; N = 2844); Figure S1: Residuals versus fitted values for the primary model (Model 2); Figure S2: LOWESS overlay on the bivariate scatter of nurse communication and 30-day readmission; File S1: STROBE checklist for cross-sectional studies [32].

## Public Involvement Statement

Patients and the public were not involved in the design, conduct, reporting, or dissemination of this research. This study used aggregate publicly reported hospital quality data and did not engage individual patients.

## Guidelines and Standards Statement

This cross-sectional study was conducted and reported in accordance with the STROBE (Strengthening the Reporting of Observational Studies in Epidemiology) guidelines for cross-sectional studies; the completed checklist is provided as Supplementary File S1.
