# Supplementary material for: Hospital-Level Nurse Communication and 30-Day Readmission in United States Acute Care Hospitals: A Cross-Sectional Centers for Medicare and Medicaid Services Hospital Compare Analysis
Source: Nurs Rep. 2026 Jun 27;16(7):222. doi: 10.3390/nursrep16070222 (PMC13415131; doi:10.3390/nursrep16070222)
Supplement: Supplementary file 1 [file nursrep-16-00222-s001.zip › nursrep-4332752-supplementary.pdf]

MDPI

nursing reports

Supplementary Materials

Hospital-Level Nurse Communication and 30-Day Readmission in United States Acute Care Hospitals Supplementary Tables

Supplementary Table S1. Quartile Analysis — Nurse Communication Quartile and 30-Day Readmission Rate (Model 2 Specification; N = 2,844)

Adjusted for hospital ownership and US Census region. Each quartile n = 711. β represents the adjusted difference in 30-day readmission rate (percentage points) vs Q1 (reference). The monotonic gradient represents a graded hospital-level association; temporal ordering between nurse communication and readmission is not established in this cross-sectional design.

Quartile	β (per 10 pp vs Q1)	95% CI	p-value
Q2	−0.193	−0.270 to −0.115	< 0.001
Q3	−0.270	−0.349 to −0.191	< 0.001
Q4 (highest)	−0.393	−0.472 to −0.315	< 0.001

Supplementary Table S2. Survey Volume Restriction — Hospitals with ≥100 Completed HCAHPS Surveys (Model 2 Specification; N = 2,660)

All other model terms (ownership, region) consistent with the primary model.

Term	β (per 10 pp)	95% CI	p-value
Nurse communication	−0.313	−0.373 to −0.253	< 0.001

Supplementary Table S3. Endogeneity Sensitivity — CMS Star Rating Sensitivity Model (N = 2,502)

The star-rating coefficient is inflated, and the nurse communication coefficient is attenuated to non-significance because the CMS star rating incorporates readmission performance (~22% weight), creating part-whole adjustment. This model is presented as a methodological illustration only.

Term	β	95% CI	p-value
Nurse communication (per 10 pp)	−0.038	−0.106 to 0.030	0.277
CMS Overall Hospital Quality Star Rating	−0.244	−0.274 to −0.215	< 0.001

Supplementary Table S4. Model Adjustment Hierarchy — Nurse Communication Coefficient Across Six Models

Model	Adjustment	N	β (per 10pp)	95% CI	p-value	R^2
M1	Nurse score only (unadjusted)	2,844	−0.259	−0.312 to −0.206	< 0.001	0.031
**M2 (Primary)**	**+ Ownership + Region**	**2,844**	**−0.289**	**−0.341 to −0.236**	**< 0.001**	**0.102**
M3	+ log Discharge volume + Survey count	2,844	−0.302	−0.358 to −0.246	< 0.001	0.103

https://doi.org/10.3390/nursrep16070222

Model	Adjustment	N	β (per 10pp)	95% CI	p-value	R^2
M4	+ State fixed effects	2,844	−0.264	−0.321 to −0.208	< 0.001	0.214
M5	+ log Bed count + Urban/rural (POS link)	2,843	−0.270	−0.330 to −0.210	< 0.001	—
**M6**	**+ Teaching intensity + Staffing density + DSH% (HCRIS link)**	**2,525**	**−0.190**	**−0.257 to −0.124**	**< 0.001**	—

All six models exclude the CMS Overall Hospital Quality Star Rating (part-whole adjustment). Model 5 attenuation relative to M2 = 6.6%; Model 6 attenuation relative to the same-subsample M5 = 26.9%.

Supplementary Table S5. HCAHPS Domain Specificity — Six Patient-Experience Composites and 30-Day Readmission All models: lm(readmit rate ~ exposure_per_10pp + ownership + region). β = percentage-point change in 30-day readmission rate per 10-pp increase in domain score; all $p < 2 \times 10^{-16}$. Sorted by |β| descending. The five non-nurse domain rows use N = 2,893; the nurse communication row uses the primary Model 2 complete-case sample (N = 2,844) consistent with Table 2. † The nurse communication row uses the primary complete-case sample (N = 2,844; Model 2, Table 2) for consistency with the main text and Figure 5; the other five domains use the domain-comparison sample (N = 2,893).

HCAHPS domain	β (per 10 pp)	95% CI	p-value
Discharge information	−0.522	−0.589 to −0.455	< 0.001
Doctor communication	−0.357	−0.410 to −0.304	< 0.001
Nurse communication †	−0.289	−0.341 to −0.236	< 0.001
Overall hospital rating	−0.214	−0.245 to −0.183	< 0.001
Communication about medicines	−0.205	−0.247 to −0.163	< 0.001
Recommend hospital	−0.190	−0.217 to −0.164	< 0.001

Supplementary Table S6. Lagged HCAHPS Sensitivity — Nurse Communication (October 2021–September 2022) versus Current 30-Day Readmission (Hybrid_HWR July 2023–June 2024)
Same Model 2 specification (exposure + ownership + region) for both rows. The lagged β (−0.308) is essentially identical to the contemporaneous β (−0.289; Δ = −0.019), indicating the contemporaneous finding is not an artefact of overlapping reporting windows.

Analysis	HCAHPS period	N	β (per 10 pp)	95% CI	p-value
Contemporaneous	Apr 2024 – Mar 2025	2,844	−0.289	−0.341 to −0.236	< 0.001
Lagged (≥9-month precedence)	Oct 2021 – Sep 2022	2,357	−0.308	−0.361 to −0.255	< 0.001

Supplementary Table S7. Structural Covariate Sensitivity — CMS Provider of Services Linkage (Model 5; N = 2,843)
Linkage by Provider Number (zero-padded to six digits); 2,843 of 2,844 hospitals matched (99.96%). Critical-access status dropped owing to perfect collinearity with the prior exclusion of critical-access facilities. Model 2 primary result (N = 2,844, β = −0.289) provided for comparison.

Term	β	95% CI	p-value

Nurse communication (per 10 pp)	−0.270	−0.330 to −0.210	< 0.001
log(staffed beds)	+0.035	0.000 to 0.070	0.050

Supplementary Table S8. Linearity Check — Centered Quadratic Term in Model 2 (N = 2,844)

Term	β	95% CI	p-value
Linear (nurse score centered, per 10pp)	−0.283	−0.336 to −0.230	< 0.001
Quadratic (nurse score centred², per (10pp)²)	+0.066	+0.010 to +0.122	0.021

Nested F-test for quadratic improvement: $F(1, 2836) = 5.32$, $p = 0.021$; $\Delta R^2 \approx 0.002$. The quadratic term is statistically significant but small in magnitude; the linear specification captures the bulk of the relationship across the empirical exposure range. See Supplementary Figure S2 for the LOWESS overlay.

Supplementary Table S9. Region × Nurse Communication Interaction (N = 2,844)

Region	Slope (β per 10pp)	Δ vs South	p (Δ vs South)
South (reference)	−0.195	—	—
Midwest	−0.284	−0.089	0.21
Northeast	−0.349	−0.153	0.062
**West**	**−0.452**	**−0.256**	**0.0003**

Joint Wald F-test for the three interaction terms: $F(3, 2834) = 4.57$, $p = 0.003$. The marginal Model 2 β (−0.289) is a region-averaged estimate; the cross-hospital gradient is steepest in the West.

Supplementary Table S10. Functional-Form Robustness — Five Specifications of the Model 2 Covariate Set (N = 2,844)

Functional form	β (nurse comm per 10pp)	Interpretation	p
Linear OLS (primary; outcome scale = %)	−0.289	reference; pp change per 10pp	< 2e-16
Log-outcome OLS	−0.019	≈ −1.87% relative per 10pp	3.3e-25
Poisson GLM with offset = log(eligible discharges)	−0.018	≈ −1.76% rate change per 10pp	3.6e-9
Quasi-binomial GLM, logit link, outcome = readmit/100	−0.023	OR per 10pp = 0.978	2.9e-26
Beta regression, logit link, outcome = readmit/100	−0.022	logit-scale, comparable to quasi-binomial	4.4e-26

All five specifications give directionally consistent and statistically significant nurse communication coefficients, with magnitude approximately 1.7–1.9% relative reduction per 10pp on the readmission scale.

Supplementary Table S11. Model 6 Structural-Confounder Sensitivity — HCRIS Linkage (N = 2,525)

Model: lm(readmit_rate ~ nurse_score_10 + ownership_cat + region + log_volume + teaching_intensity + staffing_density + DSH%).

Term	β	95% CI	p
**Nurse communication (per 10pp)**	**−0.190**	**−0.257 to −0.124**	**2.1×10^{-8}**
Teaching intensity (interns+residents FTE per bed)	+0.004	−0.060 to +0.067	0.91

Term	β	95% CI	p
**Staffing density** (FTE per bed)	**−0.014**	**−0.021 to −0.006**	**7.2×10^{-4}**
**DSH percentage** (Allowable DSH, %)	**+0.66**	**+0.40 to +0.92**	**6.3×10^{-7}**
Joint F-test for the three HCRIS terms	F(3, 2514) = 11.5	—	1.7×10^{-7}

Linkage: 88.8% of analytic sample (2,525/2,844) matched to FY2023 HCRIS Hospital Provider Cost Report by Provider CCN. Same-subsample Model 5 reference β (linked subsample only) = −0.260; Model 6 attenuation = 26.9%.

Supplementary Table S12. Sensitivity of the Nurse Communication–Readmission Association to Health-System Clustering (AHRQ Compendium of U.S. Health Systems, 2023; N = 2,844).

Primary model: lm(readmit_rate ~ nurse_score_10 + ownership_cat + region). Clustering applied via cluster-robust (CR2) standard errors and a random-intercept multilevel model.

Specification	β (per 10 pp)	95% CI	Clusters (n)
M2 OLS (primary; assumes independence)	−0.289	−0.341 to −0.236	—
M2 + CR2 cluster-robust SE (by health system)	−0.289	−0.375 to −0.202	905
M2 random-intercept multilevel (by health system)	−0.263	−0.317 to −0.208	905
M2 + CR2 cluster-robust SE (by corporate parent)	−0.289	−0.377 to −0.201	743

Linkage: 2,822 of 2,844 study hospitals (99.2%) matched the AHRQ Compendium of U.S. Health Systems, 2023, by Medicare CCN; 2,520 (88.6%) belonged to one of 581 multi-hospital health systems (largest, HCA Healthcare, 146 study hospitals); 324 hospitals were independent or unmatched and treated as singleton clusters. The intraclass correlation was 0.26 in a null model and 0.21 after covariate adjustment (about a quarter of the variance in 30-day readmission lies between systems). The unequal-cluster design effect is 7.2, driven by a few very large systems; because most hospitals are in singleton or small clusters that retain nearly full information, the realized cluster-robust standard-error inflation was modest (a factor of 1.6). The nurse communication association remained statistically significant under cluster-robust (CR2, Satterthwaite degrees of freedom) standard errors ($p = 3 \times 10^{-9}$) and essentially unchanged under a random-intercept specification. CR2, bias-reduced clustered covariance estimator (clubSandwich).

Supplementary Figures

Supplementary Figure S1. Residuals versus fitted values for the primary model (Model 2). Residuals from the ordinary-least-squares regression of the 30-day readmission rate on nurse communication score (per 10 percentage points), hospital ownership, and US Census region (N = 2,844) are plotted against fitted values; the dashed red line marks zero and the blue curve is a LOESS smoother. The approximately flat smoother and roughly symmetric scatter are consistent with the linearity assumption, while the Breusch–Pagan test indicated mild heteroskedasticity ($\chi^2 = 37.05$, df = 6, $p < 0.001$), motivating the heteroskedasticity-consistent (HC3) robust standard errors reported alongside the ordinary-least-squares estimates.

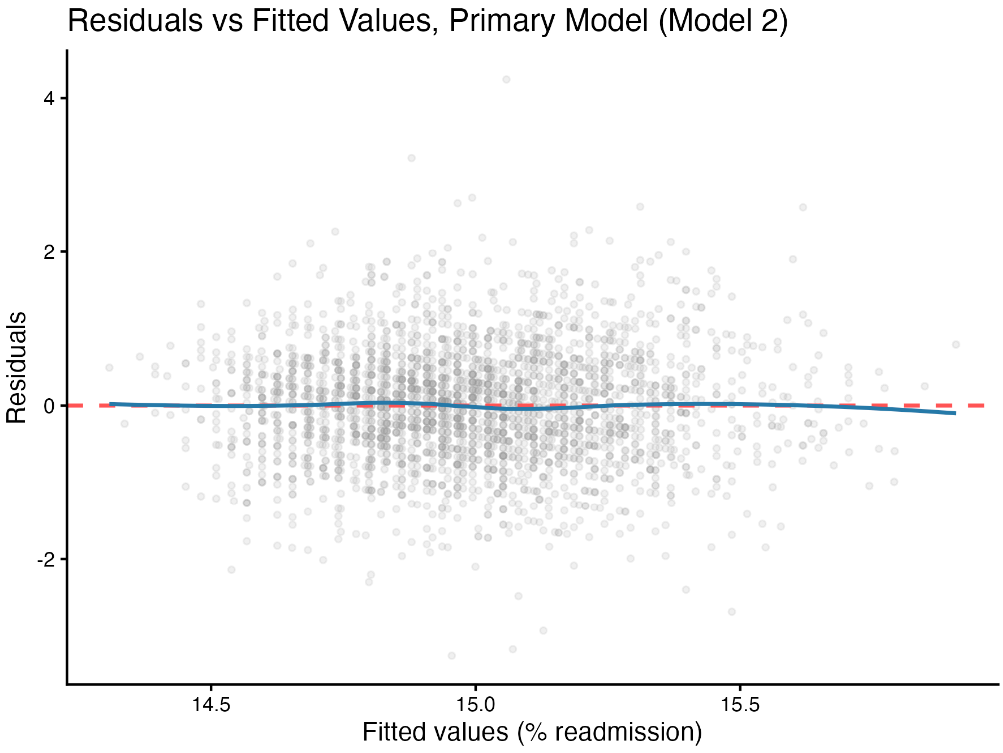

Supplementary Figure S2. LOWESS overlay on the bivariate scatter of nurse communication and 30-day readmission. Red curve = LOWESS smoother (Cleveland tricube; span = 0.5) with 95% confidence interval; dashed black = OLS fit. The two curves closely track each other across the empirical exposure range (53–97%); the quadratic term in Supplementary Table S8 is statistically significant ($p = 0.021$) but contributes $\Delta R^2 \approx 0.002$, consistent with random departure from linearity at $N = 2{,}844$ rather than substantive curvature.

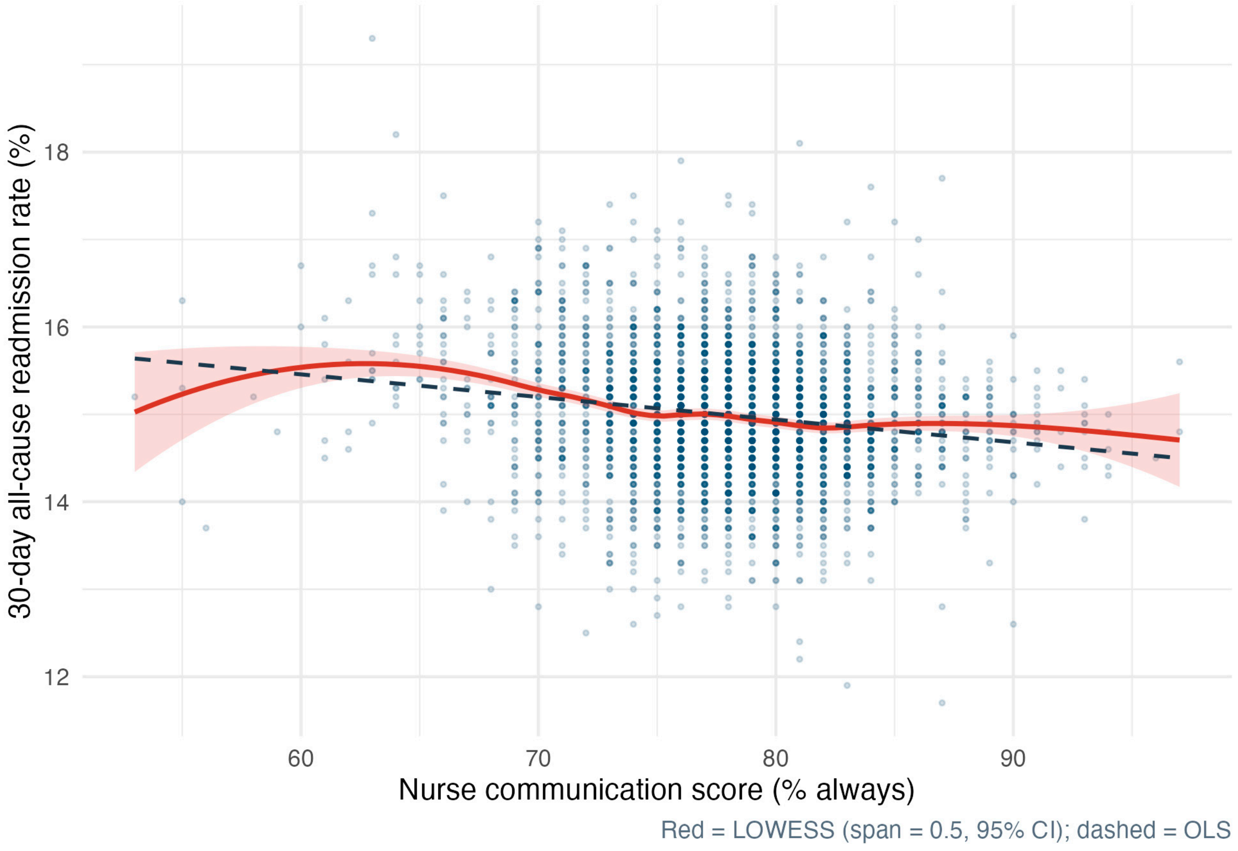

File S1. STROBE Statement—Checklist of Items That Should Be Included in Reports of Cross-Sectional Studies

Study: Nurse Communication Quality and 30-Day Hospital Readmission (nursrep-4332752).

Reference: von Elm E, Altman DG, Egger M, Pocock SJ, Gøtzsche PC, Vandenbroucke JP; STROBE Initiative. The Strengthening the Reporting of Observational Studies in Epidemiology (STROBE) statement: guidelines for reporting observational studies. Ann Intern Med. 2007;147(8):573–577.

Item No.	Recommendation	Reported in manuscript (section)
**Title and abstract**		
**1(a)**	Indicate the study's design with a commonly used term in the title or the abstract	Title: "cross-sectional secondary analysis"; Abstract: Methods
**1(b)**	Provide in the abstract an informative and balanced summary of what was done and what was found	Abstract — all four sections present
**Introduction**		
**2**	Explain the scientific background and rationale for the investigation being reported	Section 1 (Introduction), paragraphs 1–4
**3**	State specific objectives, including any prespecified hypotheses	Section 1, final paragraph
**Methods**		
**4**	Present key elements of study design at the start	Section 2.1
**5**	Describe the setting, locations, and relevant dates	Section 2.1 — US acute care hospitals; CMS Hospital Compare download date stated
**6(a)**	Give the eligibility criteria, and the sources and methods of selection of participants	Section 2.2
**6(b)**	For matched studies, give matching criteria and number of matched	N/A (no matching)
**7**	Clearly define all outcomes, exposures, predictors, potential confounders, and effect modifiers	Sections 2.3–2.5
**8**	For each variable of interest, give sources of data and details of methods of assessment	Section 2.3 (exposure), 2.4 (outcome), 2.5 (covariates)
**9**	Describe any efforts to address potential sources of bias	Section 2.6 (sensitivity analyses); Section 4.4 (Limitations)
**10**	Explain how the study size was arrived at	Section 3.1 — all eligible hospitals included; no a priori sample-size calculation needed for secondary analysis of an existing population
**11**	Explain how quantitative variables were handled in the analyses	Section 2.6 — nurse score continuous (primary) and quartile (sensitivity); readmission continuous
**12(a)**	Describe all statistical methods, including those used to control for confounding	Section 2.6 — OLS regression with stated covariates
**12(b)**	Describe any methods used to examine subgroups and interactions	Section 2.6 — quartile sensitivity; ≥100 surveys sensitivity

**12(c)**	Explain how missing data were addressed	Section 2.2 — hospitals with missing exposure/outcome/covariates excluded; exclusion numbers in the Table 1 flowchart
**12(d)**	If applicable, describe analytical methods taking account of sampling strategy	N/A — all eligible hospitals included
**12(e)**	Describe any sensitivity analyses	Sections 2.6 and 3.4
**Results**		
**13(a)**	Report numbers of individuals at each stage of study (potentially eligible, examined for eligibility, confirmed eligible, included)	Section 3.1; Table 1
**13(b)**	Give reasons for non-participation at each stage	Sections 2.2 and 3.1 — exclusion criteria stated
**13(c)**	Consider use of a flow diagram	Figure 1 (STROBE-style sample flow diagram)
**14(a)**	Give characteristics of study participants and information on exposures and potential confounders	Table 1
**14(b)**	Indicate number of participants with missing data for each variable of interest	Reported in Section 3.1
**15**	Report numbers of outcome events or summary measures	Section 3.1 and Table 1
**16(a)**	Give unadjusted estimates and, if applicable, confounder-adjusted estimates	Table 2 — adjusted estimates; Pearson r in Section 3.2 (unadjusted)
**16(b)**	Report category boundaries when continuous variables were categorized	Section 2.6 — quartile cut-points reported in output
**16(c)**	If relevant, consider translating estimates of relative risk into absolute risk for a meaningful time period	Section 4.1 — absolute readmission difference between Q1 and Q4 stated
**17**	Report other analyses done — e.g., analyses of subgroups and interactions, and sensitivity analyses	Section 3.4; Supplementary Tables S1–S2
**Discussion**		
**18**	Summarise key results with reference to study objectives	Section 4.1
**19**	Discuss limitations of the study, taking into account sources of potential bias or imprecision; discuss both direction and magnitude of any potential bias	Section 4.4
**20**	Give a cautious overall interpretation of results considering objectives, limitations, multiplicity of analyses, results from similar studies, and other relevant evidence	Sections 4.2–4.4
**21**	Discuss the generalisability (external validity) of the study results	Section 4.4 — restricted to Medicare/Medicaid-participating hospitals
**Other information**		
**22**	Give the source of funding and the role of the funders for the present study and, if	Funding and Declarations section

	applicable, for the original study on which the present article is based	

Note: STROBE items are reporting guidelines, not rigid prescriptions; not all items apply to a cross-sectional secondary analysis of publicly available aggregate data.
